# How are microbes helping end hunger?

**DOI:** 10.1111/1751-7915.14432

**Published:** 2024-03-11

**Authors:** Patricia Bernal

**Affiliations:** ^1^ Departamento de Microbiología, Facultad de Biología Universidad de Sevilla Seville Spain

## Abstract

This article explores the potential of microbiology to positively impact all aspects of the food supply chain, improving the quantity, quality, safety, and nutritional value of food products by providing innovative ways of growing, processing, and preserving food and thus contributing to Zero Hunger, one of the Sustainable Development Goals (SDGs) of the United Nations.

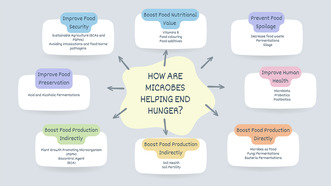

Hunger is a cruel reality that affects millions of people around the world and could worsen in the coming years due to the continuous growth of the population and the limitations of our planet's resources. Feeding a population that is expected to exceed 9 billion by 2050 is extremely complex and requires not only an adequate and sustainable food supply but also a collective effort from different sectors of society. This collaborative effort should involve key stakeholders, including politicians to inspire social and cultural changes and scientists to provide technological innovations. Social changes such as promoting education on proper nutrition practices or encouraging healthy lifestyles are as important as ensuring an efficient, secure and sustainable food system. The efficiency of the system can be improved by reducing lost and waste, minimizing water use and improving nutrition quality. The food system must also ensure the health of humans and animals by preventing foodborne diseases and intoxications through improved sanitary conditions. Importantly, the food supply system must be sustainable to protect not only the well‐being of individuals but also the overall health of our planet. The current way of producing food is unsustainable because it requires large amounts of energy and water, contributes to climate change and degrades ecosystems. It is essential that the food supply system can operate without causing further damage to the planet and preserving its biodiversity. This can be achieved, among other actions, by reducing greenhouse gas emissions and avoiding recalcitrant contaminants and land deforestation. In addition to the growing population, climate change itself poses a significant threat to the challenge discussed. Climate change is responsible for an increase in the number of extreme weather events, changes in temperature and precipitation patterns and the frequency of droughts and floods. All of these events disrupt crop yields, compromise food security and contribute to the spread of pests and diseases.

As microbiologists, we are responsible for delivering innovative solutions to these challenges, since microorganisms, although historically ignored and underestimated, have great potential for many positive contributions. Microorganisms are valuable allies that help maintain and improve ecosystems and thus counteract the adverse impacts of intensive farming and climate change, that is, reduce the environmental footprint of agriculture. In addition, they can improve the productivity and efficiency of all aspects of the food system, including production, distribution and consumption, and more importantly, food security.

This article explores the potential of microbiology to positively impact all aspects of the food supply chain, improving the quantity, quality, safety and nutritional value of food products by providing innovative ways of growing, processing and preserving food and thus contributing to Zero Hunger, one of the Sustainable Development Goals (SDGs) of the United Nations.

## HOW MICROBIOLOGY CAN BOOST FOOD PRODUCTION

### Indirectly: Soil health and plant growth‐promoting microorganisms

Healthy and fertile soils foster optimal conditions for the growth of crops, which is fundamental for the sustainability of the food supply chain, but many anthropogenic activities cause significant levels of soil degradation. To address the current soil crisis, different microbial tools have been developed to improve soil fertility, facilitate the decomposition of organic matter (Sáez‐Sandino et al., 2023), promote nutrient cycling, detoxify pollutants (Jayaramaiah et al., 2022) and support the overall resilience of the ecosystems. The importance of maintaining soil health and the positive influence of microorganisms in addressing this challenge has been discussed by Timmis and Ramos (2021), who advocate for the creation of a soil health system to proactively prevent soil loss.

Importantly, microorganisms can directly improve vegetable nutritional levels, which would improve nutrient intake without requiring increased consumption (Goicoechea & Antolín, 2017). They can also promote food production by stimulating the growth of plants, the primary producers of the food chain. Plants, beyond their nutritional value, provide the raw materials for numerous food products, including animal agriculture. Microorganisms with the capacity to improve the growth of plants and, more importantly, crops are known as plant growth‐promoting microorganisms (PGPMs). To foster plant growth, many PGPMs can produce phytohormones, such as ethylene, cytokinin, gibberellin, auxin or salicylic acid (Nascimento et al., 2021) that stimulate physiological processes in plants, including cell division and root development. Other PGPMs can form symbiotic relationships with plants, contributing to better nutrient uptake by solubilizing and mobilizing essential nutrients such as phosphorus or nitrogen (Zhao et al., 2023), and increasing tolerance to environmental stress. For example, the symbiotic relationship between mycorrhizal fungi and plants provides the plant with a limited and important nutrient, phosphorus (Shi et al., 2023). Similarly, nitrogen‐fixing (NF) bacteria form symbiotic relationships with certain plant (leguminous) roots and provide nitrogen. NF bacteria promote the formation of a new organ in the root, the nodule, where they fix atmospheric nitrogen (N_2_) in a form that can be assimilated by plants such as ammonia. The nitrogen fixation process not only enhances the growth of the plant but also the fertility of the soil, reducing the need for synthetic nitrogen fertilizers (Jhu & Oldroyd, 2023). PGPMs can also contribute to enhance the resilience of plants under challenging environmental conditions associated with climate change, including resilience to drought, floods, extreme temperatures and other adverse conditions (Li et al., 2023). For example, certain PGPMs produce osmoprotectants that help plants cope with water scarcity during drought conditions (Fadiji et al., 2022). Other PGPMs help plants survive in oxygen‐deprived conditions by improving oxygen availability in waterlogged soils, a common consequence of flooding (Ali & Kim, 2018). They can mitigate temperature‐related stress, which impacts plant growth, inducing the production of heat‐shock proteins in plants, and helping them withstand temperature fluctuations and heat waves (Seth & Sebastian, 2024). Some PGPMs help plants cope with high soil salinity by facilitating the removal of excess salts from plant tissues, a problem also exacerbated by changing climate conditions (Ali et al., 2023).

In general, the use of PGPMs can significantly improve the resilience of plants to environmental stressors such as nutrient limitations, drought conditions, flooding, extreme temperatures and high soil salinity. These interactions improve plant growth and soil fertility, reducing the dependence on synthetic fertilizers and mitigating the negative effects of climate change on crops.

### Indirectly: Biological control agents

Although most microorganisms are beneficial from a human point of view with the potential to stimulate the food system, some pathogenic microorganisms can cause severe economic losses during the production phase. Phytopathogens are responsible for causing diseases in plants that could damage edible parts or reduce overall crop production. The type III secretion system (T3SS) is one of the most important virulence factors in these microorganisms and has been extensively studied for years. The T3SS is a specialized protein delivery system that certain bacteria use to inject effector proteins directly into the cells of the host plant. These effectors manipulate the host's cellular processes, enabling the pathogen to establish infection and evade the plant's defence mechanisms (Schreiber et al., 2021). Given its pivotal role in the pathogenicity of many phytopathogens, T3SS has become a major target for the development of inhibitors. These inhibitors have the potential to disrupt the pathogen's ability to inject effectors, thereby limiting its ability to cause disease and thus protecting crops from the damaging effects of phytopathogens (Yuan et al., 2020).

On the contrary, beneficial microorganisms such as PGPMs can act as natural agents to protect crops by suppressing the growth of harmful pathogens, that is, controlling pests and diseases. They are known as biological control agents (BCAs) or biopesticides and are a great alternative to chemical pesticides to promote sustainable farming practices. BCAs have in common the ability to eliminate or control plant pathogen growth, but they can use a variety of different strategies to do so. Certain microorganisms can induce systemic resistance in plants by activating natural defence pathways and preparing plants to respond more efficiently to subsequent pathogen attacks. The application of ISR‐inducing microorganisms can enhance the overall resilience of plants to diseases (Pieterse et al., 2014; Salwan et al., 2023). BCAs can limit the growth of the plant pathogen by competing for the same resources (nutrient competition), and importantly for iron by using siderophores, that is, small, high‐affinity iron chelating compounds. Since many pathogenic bacteria require iron for their growth, BCA‐produced siderophores are a good strategy to limit their growth and virulence and reduce the incidence of plant diseases (Ahmed & Holmström, 2014). Other BCAs use strategies directly involved in interbacterial competition, killing or inhibiting the growth of pathogens. For example, some microorganisms produce antimicrobial compounds, such as antibiotics or antimicrobial peptides (AMP), which are secreted into the environment and inhibit the growth of pathogens (Roca & Matilla, 2023). Some BCAs can produce hydrogen cyanide (HCN), a secondary metabolite released by certain bacteria that interferes with the cellular respiration of soilborne pathogens, inhibiting their growth and causing cell death (Barahona et al., 2011). In general, these methods offer non‐contact approaches to inhibit the growth of pathogens in the environment. In contrast, some bacteria possess contact‐dependent complex molecular machineries that inject toxic proteins directly into rival bacterial or eukaryotic cells. The best characterized is the Type VI Secretion System (T6SS) (Allsopp & Bernal, 2023) present specifically in gram‐negative bacteria. The T6SS has been described as a potent mechanism of biocontrol that enables certain bacteria to target and kill pathogens in their ecological niches (Bernal et al., 2017, 2021; Durán et al., 2021), and the same holds true for the T4BSS (Purtschert‐Montenegro et al., 2022). Similarly, predatory microorganisms have been recognized as valuable biocontrol agents (Zhang et al., 2023). These microorganisms produce enzymes to degrade the cell walls of target organisms or use specialized structures to capture, penetrate and consume their prey. In particular, Myxobacteria and *Bdellovibrio and like organisms* (BALOs) exhibit a broad predation spectrum in plant pathogens, making them good candidates for biocontrol applications (Zhang et al., 2023). Likewise, viruses that specifically infect and kill phytopathogenic microorganisms can be used in biocontrol to reduce the population of these harmful organisms. This novel approach minimizes the impact on non‐target organisms and is considered a promising biocontrol strategy currently underused mostly because of regulatory obstacles (Wagemans et al., 2022). Many phytopathogenic bacteria exhibit social behaviour that promotes infections, including the formation of biofilms that allow these bacteria to adhere to biological surfaces as the first step of infection. Some BCAs can disrupt the formation of biofilms by producing compounds that interfere with their adhesive properties, making it difficult for pathogens to cause infections. Pathogenic bacteria can coordinate the gene expression of these ‘social’ virulence factors, for example, biofilm formation or T3SS, at the population level by using a Quorum Sensing (QS) communication system. Some BCAs can interfere with pathogen's QS by a mechanism known as Quorum Quenching (QQ) (Liu et al., 2023) that inhibits the coordinated expression of virulence factors. Other signalling molecules that can be released by biocontrol agents are volatile organic compounds (VOCs). These molecules can interfere with spore germination, inhibit pathogen growth or induce systemic resistance in plants, and therefore could be exploited for pest control in sustainable agriculture (Almeida et al., 2023).

All these mechanisms contribute to the diverse arsenal of strategies employed by PGPMs and BCAs. Research in these fields continues to uncover new insights into our understanding of microbial interactions with plants, offering opportunities to optimize these relationships for the benefit of crop production, disease management practices and ecosystem health. A recent example of the new technologies that are being developed in this field is the seed biopriming system. This novel microbial inoculant technique combines effective BCAs and physiological aspects of the seed, such as hydration, to improve metabolism activity and mitigate the common limitations of bioinoculants (Singh, Vaishnav, et al., 2023). In fact, seed biopriming has been shown to increase the protection of seeds against soil‐borne pathogens and soil pollutants, for example, salts and heavy metals, while promoting germination rate and uniformity, improving not only the primary productivity but also soil health (Singh, Vaishnav, et al., 2023).

In the last decade, the promising application of these novel practices in agriculture has gained attention as sustainable and environmentally friendly approaches to improve crop productivity and its importance is expected to continue to grow in the future.

### Direct role: Microbes as food and fermentation processes

Fungi that form fruiting bodies such as mushrooms and others alike are an excellent source of direct microbial food that is easily cultivated and cooked. In addition, in recent years, microbial products from fungi mycelium, such as Mycoprotein or Quorn (brand), have hit the market. Quorn is a protein‐rich food ingredient produced from *Fusarium venenatum* that is often used as the primary component of vegetarian ‘meat’ because it offers a meaty texture and a suitable protein content (Banks et al., 2022). Fungi mycelia from species such as *Pleurotus ostreatus* (oyster mushroom) and *Ganoderma lucidum* (reishi mushroom) are also used in the production of meat alternatives or snack products, such as mushroom chips or mushroom jerky. Furthermore, fungal mycelium extracts are also used in the production of functional beverages, including teas and elixirs, with potential health benefits. In recent decades, photosynthetic microorganisms like the cyanobacterium *Spirulina* have been cultivated for their nutritional value containing essential amino acids, vitamins and antioxidants. These bacteria hold the promise of becoming a major food source in the future (García et al., 2017) as an alternative to animal protein. Moreover, some species of filamentous cyanobacterium *Nostoc* are commonly consumed in certain Asian cultures, particularly in China, and it is also a good food source due to its high protein content.

The cultivation of fungi or photosynthetic microbes typically requires less land, water and resources compared to traditional agriculture, making them a great sustainable food source. However, the taste and acceptance of these products in new cultures can be a challenge, and many efforts are ongoing to improve palatability.

Microorganisms are also essential for various processes directly involved in food production, especially fermentation, a traditional method that has been used for centuries. Biochemically speaking, it is a metabolic process in which microorganisms break down complex compounds into simpler ones, producing by‐products that contribute to the taste, texture, nutritional content and bioactive properties of foods (Kiczorowski et al., 2022). Examples include the fermentation of milk to produce yoghurt, cheese or kefir; the fermentation of cabbage to produce sauerkraut; the fermentation of cereals for the production of bread or beer or the fermentation of grapes to produce wine, vinegar, champagne or spirits such as brandy or grappa. The fermentation of plant‐based dairy is a growing field that has emerged as an important alternative to the fermentation of animal products (Harper et al., 2022). Conscious manipulation of the microorganisms involved in these processes could improve not only consistency but, more importantly, flavour quality.

Given the extensive list of microorganisms involved in fermentation and food products resulting from this process, presenting them all here would be impractical, and readers can refer to fantastic reviews including (Tamang et al., 2020). Instead, we highlight key food products and producer microorganisms of great interest to humans, due to their nutritional value and profound impact on human culture.

#### Fungi fermentations

*Saccharomyces* cerevisiae, also known as baker's yeast, is a fungus commonly used in baking and brewing. *Saccharomyces* in Greek means ‘sugar fungus’ since during fermentation, this fungus uses sugars and, as a by‐product, produces alcohol. Alcohol is a molecule without biological function and is extremely toxic to most microorganisms, except for this resistant fungus that uses it as a chemical weapon to eliminate competitors. This fungus has been widely studied for decades due to its involvement in food production, and many revisions are available in the literature (Gänzle, 2022).

The role of *Saccharomyces* and other yeasts is also essential in the first steps of cocoa fermentation and is directly involved in the development of the cocoa flavour. Cocoa beans are very bitter and acidic and must go through a correct fermentation process to lose bad taste and obtain good quality chocolate. Up to 45 yeast genera have been identified in cocoa fermentation in different countries including *Saccharomyces*, *Pichia*, *Candida*, *Hanseniaspora*, *Torulaspora*, *Issatchenkia* and *Saccharomycopsis* (Schwan et al., 2023). *Saccharomyces* have also been involved in the spontaneous fermentation of coffee beans together with other microorganisms that are part of a complex microbiota present in the fruit. This complex microbiota formed by yeasts, filamentous fungi and bacteria can vary depending on the environmental conditions, coffee varieties, fruit maturation, season, altitude, temperature and processing methods affecting the quality of this beverage (Schwan et al., 2023). Similarly, *Saccharomyces cerevisiae* and other fungi, including *Penicillium roqueforti*, *Penicillium camemberti* and *Geotrichum candidum*, have been used for centuries to produce cheese and *Penicillium nalgiovense* and *Penicillium salami* to dry‐cure meat, such as salami (Ropars & Giraud, 2022). Interestingly, for food biotechnology, distant fungal lineages have been described to be domesticated for specific fermentation processes such as cheese or meat making by parallel adaptation events. These events lead to phenotypic convergence important for fermentation processes such as lipolysis, proteolysis, volatile compound production and competitive ability against food spoilers and, at the same time, are responsible for the degeneration of unused traits such as toxin production (Ropars & Giraud, 2022). Fungi are also responsible for the production of certain alcoholic beverages, such as sake, that result from rice fermentation by *Aspergillus oryzae*. This fungus, together with *Aspergillus sojae*, is also involved in the first steps of the fermentation of soybeans and wheat to produce a widely used fermented condiment, soy sauce (Luh, 1995). In the second step of production, the mould mixture named koji undergoes yeast fermentation facilitated by *Zygosaccharomyces rouxii* and *Candida* sp. along with lactic acid fermentation (Luh, 1995), a process described below.

#### Lactic acid bacteria (LAB)

The fermentation of milk by lactic acid bacteria is a well‐understood process in which caseins in milk undergo proteolysis into peptides, and amino acids and carbohydrates are transformed into lactic acid. These processes are fundamental to developing the flavour and texture of fermented dairy products like cheese, kefir and yoghurt. Moreover, LAB produce compounds such as acetoin and diacetyl that are responsible for the characteristic tastes of cheeses and other dairy products. Not only LAB but also yeasts like *Geotrichum candidum* can produce a mixture of alcohols, fatty acids and other compounds responsible for the fruity aroma characteristic of cheeses such as brie or camembert. The microbial population involved in these processes plays a critical role in preserving the quality and distinctive flavours of different products. For example, a study on cheese flavour has revealed the crucial role of *Streptococcus thermophilus* in the growth of another bacterium, *Lactococcus cremoris*, which has a significant impact on the flavour palette because it limits the formation of unpleasant compounds (Melkonian et al., 2023). The production of other dairy products, such as kumis (or koumiss), an alcoholic beverage made from fermented mare's milk, involves both lactic acid and alcoholic fermentation. The first step is the lactic acid fermentation of milk by different *Lactobacillus* species resulting in an acidic beverage that then undergoes an alcoholic fermentation by *Saccharomyces lactis* and other fungi, including *Kluyveromyces lactis*, which turns it into a carbonated and mildly alcoholic drink. (Behera et al., 2017; Tesfaye et al., 2019). In addition to dairy products fermentation, LAB including several species of *Leuconostoc*, *Lactobacillus*, and *Weissella* are used to ferment vegetables such as napa cabbage or Korean radish to produce traditional Korean kimchi. Similarly, the fermentation of raw cabbages by LAB produce sauerkraut, cucumbers are transformed into cucumber pickles and olives are fermented by yeasts and LAB to remove bitterness and make them palatable. In recent years, a fermented tea beverage known as kombucha has become a fashionable and healthier alternative to carbonated beverages and can be found in many supermarkets around the world. The fermentation occurs by a symbiotic culture of bacteria and yeast (SCOBY) consisting of LAB, acetic acid bacteria (AAB) and yeast, which metabolize the sugar and tea components. This process results in a naturally carbonated beverage, with a flavour that combines sweet and sour, antioxidants and vitamins and with trace amounts of alcohol (Diez‐Ozaeta & Astiazaran, 2022).

The fermentation process is the most popular food processing technique not only because it allows an effective preservation of food but also because it increases the nutritional and functional values of these food products.

## HOW MICROBIOLOGY CAN BOOST THE NUTRITIONAL VALUE OF FOOD

Microorganisms are instrumental in the synthesis of essential nutrients and vitamins during food production processes. For instance, certain bacteria such as LAB contribute to the synthesis of B vitamins in fermented foods, for example, yoghurt or kombucha, improving their nutritional profile and providing health benefits for consumers (Capozzi et al., 2012). Microorganisms can also be used in the production of specific food ingredients and additives. For example, *Aspergillus niger* produces citric acid, a compound that is widely used in the food industry as an acidulant to provide a sour taste. Similarly, *Corynebacterium glutamicum* is responsible for the production of monosodium glutamate (MSG), a common food additive used to enhance the taste of many dishes. Microorganisms can also be a source of food colouring, for example, *Spirulina* and other cyanobacteria produce green and blue pigments, named phycocyanin, commonly used in the food industry. Other bacteria like *Agrobacterium aurantiacum* or *Paracoccus carotinifaciens* produce astaxanthin, a pink/red pigment used in animal products to enhance the food's natural colour. Fungi like *Mucor circinelloides*, *Neurospora crassa* and *Phycomyces blakesleeanus* produce β‐carotene (yellow/orange) that is used in the production of pastry, cream, cheese and ice cream (Singh, Pandey, et al., 2023).

All of these microbial pigments have associated a variety of health benefits, including antioxidant, anticancer and anti‐inflammatory properties that can have a significant impact on human health.

## HOW MICROBIOLOGY CAN SECURE FOOD SECURITY AND PRESERVATION AND IMPROVE HUMAN HEALTH

Directly involved in our health are the microorganisms that form our microbiota, especially gut microbes. They are responsible for proper digestion of food and absorption of nutrients, contribute to vitamin synthesis and the development of lymphoid structures in the gastrointestinal tract and act as a protective barrier against pathogens competing for nutrients and space (Yi et al., 2023). Likewise, probiotics, that is, microorganisms known for their positive effects on our digestive system, can play an important role in human health. Bacteria from the genera *Lactobacillus* and *Bifidobacterium*, and certain yeast strains like *Saccharomyces boulardii* are the best known but others like *Akkermansia* shine as promising novel probiotics (Zhang et al., 2019). In recent years, a new concept referred to as postbiotics, that is, the metabolites or by‐products produced by probiotic microorganisms, has received a lot of attention. These by‐products offer multiple advantages over probiotics, especially in terms of stability. The increased stability allows postbiotics to have a shelf life longer than that of live microorganisms and to be more easily handled, transported and incorporated into various food and supplement products without the need for refrigeration. Postbiotics exert their beneficial effects through various mechanisms, such as modulating immune function, promoting integrity of the intestinal barrier and reducing inflammation. Consequently, postbiotics can have a huge impact on diseases such as obesity, which is currently a major risk factor for morbidity and mortality worldwide. For instance, a recent study has shown that lipoteichoic acid (LTA) from *Bifidobacterium animalis* is a postbiotic capable of improving obesity biomarkers by reducing fat levels, even under hyperglycaemic conditions (Balaguer et al., 2022). Therefore, LTA can be used as a therapeutic or preventive agent for diseases associated with metabolic disorders. In a similar line, extracts of fungal mycelium, particularly mushrooms used in traditional medicine such as Cordyceps (*Ophiocordyceps sinensis*), Reishi (*Ganoderma lucidum*) and Lion's Mane (*Hericium erinaceus*), are used in the production of dietary supplements, marketed for their potential health benefits, including immune support and cognitive enhancement (Łysakowska et al., 2023). Indirectly, BCAs and PGPMs help protect against pathogenic microbes and enhance plant growth, reducing/avoiding the use of chemical pesticides and fertilizers. Thus, the consumption of food produced through these sustainable practices ensures a lower presence of chemicals in our food supply, that is, healthier products.

In contrast, microbial contamination can pose a challenge in the food industry, since pathogens can contaminate food products at any stage of the food chain. Bacteria such as *Salmonella* or *Listeria* represent a serious public health concern, as they can cause severe illnesses when consumed. *Salmonella* is associated with undercooked eggs, poultry or meat while *Listeria* is linked with ready‐to‐eat foods, including deli meats, soft cheeses and smoked fish. Unpasteurized dairy products are related to diseases caused by *Salmonella*, *Listeria* and *Brucella*, the causal agent of brucellosis, a disease that could cause abortion in pregnant women. In other cases, contamination of food by microorganisms does not result in human diseases but in intoxications due to the secretion of toxins by microorganisms. For example, *Clostridium perfringens*, whose spores can be found in the intestines of poultry, secretes an enterotoxin (CPE) responsible for the symptoms of *C. perfringens* food poisoning. Not only bacteria but also numerous fungi can induce food intoxication through the production of toxins exemplified by *Claviceps purpurea*, commonly known as ergot. This fungus infects certain cereal crops, particularly rye, and produces toxic compounds from the alkaloid family. Consumption of grains contaminated with ergot can lead to ergotism, a disease characterized by severe symptoms like hallucinations, convulsions and gangrene.

Therefore, while embracing sustainable agricultural practices and harnessing the benefits of beneficial microorganisms, it is crucial to address and mitigate the risks associated with microbial contamination to ensure a safe and healthy food supply for consumers and to avoid food spoilage.

## HOW MICROBIOLOGY CAN PREVENT FOOD SPOILAGE

Substantial amounts of food are lost or wasted at various stages of the supply chain, from production to processing, distribution and consumption. Two main causes are responsible for food spoilage, the presence of phytopathogens that directly ruin the collected product, or microorganisms that produce toxins that accumulate in food and cause intoxications when consumed. Addressing these inefficiencies in the food system and reducing waste is critical to maximize the impact of available resources. These mechanisms have been previously named; that is, BCAs can help prevent food spoilage by inhibiting the growth of pathogens or toxins‐producing microorganisms during the phases of production, processing and distribution, extending the shelf life of perishable items. Similarly, microorganisms responsible for fermentation processes preserve food products, reducing perishability and contamination with pathogens and mycotoxins (Adebo et al., 2019) due to the inhibitory effect of toxic compounds produced as by‐products such as alcohol or acids (Guo et al., 2023). An interesting example of animal food (forage) preservation is silage made from green crops, such as grass or corn, that are stored and fermented under airtight conditions. The absence of oxygen promotes fermentation by lactic acid bacteria, which produce an acidic environment that helps preserve forage for months, preventing spoilage and decay. Furthermore, current knowledge about the use of LAB in crop silage indicates that the fermentation process improves the digestibility of the silage by livestock, and is a vehicle for the delivery of probiotic substances that can expand animal health (Guo et al., 2023).

On a different line, microbes can be directly involved in waste management within the food industry. They can be used in processes such as composting and anaerobic digestion to convert organic waste into valuable products such as fertilizers and biogas, promoting sustainability and reducing environmental impact (Rastogi & Barapatre, 2021; Zhang et al., 2022).

In conclusion, microbes are indispensable allies in the intricate web of food production and their importance is expected to continue increasing. They enhance manufacturing, improve food properties, prevent spoilage and pathogenicity and contribute to human health. In this way, they are crucial to ensuring the sustainability and efficiency of the entire food supply chain. Importantly, recognizing and harnessing the power of microbes in food production is the key to addressing global food security challenges and promoting a more sustainable and resilient food system.

**FIGURE 1:**
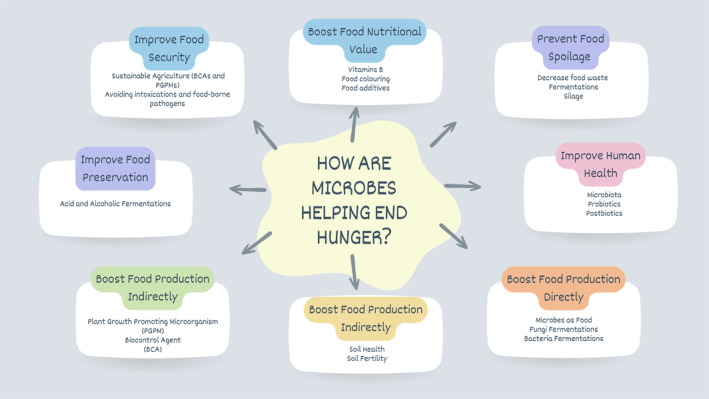
Representation of different stages where microorganisms play a crucial role in sustaining global food production.

## AUTHOR CONTRIBUTION

Patricia Bernal conceived the idea for this article, conducted all the necessary literature review, and wrote the entire manuscript.

## CONFLICT OF INTEREST STATEMENT

The author declares that there is no conflict of interest.
